# Slim but strong: A powerful ally, the over‐the‐wire microelectrode catheter

**DOI:** 10.1002/joa3.12894

**Published:** 2023-06-27

**Authors:** Tatsuya Hayashi, Hideo Fujita

**Affiliations:** ^1^ Division of Cardiovascular Medicine Saitama Medical Center Jichi Medical University Saitama Japan

The coronary sinus (CS) catheter, which provides left atrial and left ventricular potentials and also serves as an anatomical marker, is indispensable in modern EPS. However, inserting CS catheters can sometimes be challenging due to anatomical anomalies or the presence of left ventricular (LV) leads. In this context, Okada et al. demonstrated the usefulness of the novel over‐the‐wire 2.7‐Fr microcatheter (EPstar Fix AIV; Japan Lifeline), which has a lumen and can be inserted into the CS with a 0.014‐inch wire leading. While the previous use of another thin 2Fr electrode catheter (EPstar Fix, Japan Lifeline) has been reported,^1^ it should be noted that this “lumenless” catheter is a regular electrode catheter, and cannot be inserted over a guidewire. Consequently, its insertion can be challenging and less user‐friendly in cases of curved veins, such as branches of the CS.

Conversely, the current over‐the‐wire 2.7Fr microcatheter can navigate through tortuous veins using a 0.014‐inch guidewire through its lumen, enabling successful insertion even in the curved veins. Moreover, prior angiography of the coronary sinus using a guiding catheter enables precise vein selection for catheter insertion. This novel over‐the‐wire catheter was initially designed with the specific intention of being used in the distal site of CS, such as the anterior interventricular vein (AIV), as indicated by its name.^2^

However, the present study emphasizes the broader applications of this catheter. The authors propose using the over‐the‐wire catheter in cases where insertion into the main trunk of the CS is challenging or carries a risk of LV lead dislodgement, rather than for deep insertion into the CS distal. This catheter facilitates safe insertion in scenarios involving CS stenosis, possibly resulting from anatomical anomalies, post‐catheter ablation involving the CS, or challenging catheter insertion after LV lead placement. The small diameter of this microcatheter also minimizes the risk of LV lead dislodgement during and after insertion.

Furthermore, while this study primarily focuses on placing the catheter in the main trunk of the CS, there are additional advanced applications to consider. Although initially intended for insertion into the distal CS, such as the AIV, this catheter also allows selective insertion into other CS branches, including the lateral vein or middle cardiac vein (MCV), which may serve as the origin of premature ventricular contractions (PVC) or ventricular tachycardia (VT) originating from the coronary sinus.^3^

The novel over‐the‐wire 2.7‐Fr microcatheter features eight electrodes with a diameter of 0.65 mm, facilitating accurate electrical recordings despite its slim profile from both the superior vena cava (SVC) and inferior vena cava (IVC). This “Slim & Strong” catheter, characterized by its enhanced safety and practicality, is promised to offer considerable clinical utility, thus resulting in advancements in the diagnosis and treatment of EPS and catheter ablation procedures.

## CONFLICT OF INTEREST STATEMENT

No conflicts of interest related to this topic.

## References

[1] Fujisawa T , Kimura T , Nakajima K , Nishiyama T , Katsumata Y , Aizawa Y , et al. Importance of the vein of Marshall involvement in mitral isthmus ablation. Pacing Clin Electrophysiol. 2019;42:617–24. 30779354 10.1111/pace.13640

[2] Shirai Y , Goya M , Sasaki T , Nagasawa R , Toya C , Hayasaka K , et al. Usefulness of the over‐the‐wire microelectrodes catheter in treatment of ventricular arrhythmia arising from the left ventricular summit. Pacing Clinical Electrophis. 2022;45:1141–50. 10.1111/pace.14542 35665518

[3] Mountantonakis SE , Frankel DS , Tschabrunn CM , Hutchinson MD , Riley MP , Lin D , et al. Ventricular arrhythmias from the coronary venous system: prevalence, mapping, and ablation. Heart Rhythm. 2015;12:1145–53. 25766774 10.1016/j.hrthm.2015.03.009

